# Cinnamaldehyde supplementation in sows and their offspring: effects on colostrum and milk composition, performance, redox status and intestinal health

**DOI:** 10.1186/s40104-025-01212-x

**Published:** 2025-06-03

**Authors:** Junqi Jin, Shiya Liu, Qiang Zhou, Zhengfeng Fang, Yan Lin, Shengyu Xu, Bin Feng, Yong Zhuo, Hefeng Luo, Xiuming Liu, De Wu, Lianqiang Che

**Affiliations:** 1 Key Laboratory for Animal Disease-Resistant Nutrition of China Ministry of Education, Key Laboratory for Animal Disease-Resistant Nutrition of Sichuan Province, Animal Nutrition Institute, Chengdu, Sichuan 61130 China; 2 Dekon Food and Agriculture Group, Chengdu, Sichuan 610225 China; 3 Department of Gynaecology, Guangzhou Liwan District Hospital of Traditional Chinese Medicine, Guangzhou, Guangdong 510140 China

**Keywords:** Antioxidant capacity, Cinnamaldehyde, Intestinal health, Microbiome, Piglets, Sow

## Abstract

**Background:**

Maternal nutrition significantly influences offspring development. This study investigated the effects of maternal or post-weaning cinnamaldehyde (CA) supplementation in sows and their offspring on reproductive performance and health. Sixty sows, selected based on body condition score and parity, were randomly allocated to control or CA (500 mg/kg) diets from d 107 of gestation to d 24 of lactation. At weaning, 128 piglets were assigned to four groups (*n* = 8) based on weight and source litter for a 21-d experiment. The four groups were CON-CON (both sow and piglet on CON), CON-CA (sow on CON, piglet on CA), CA-CON (sow on CA, piglet on CON), and CA-CA (both sow and piglet on CA).

**Results:**

Maternal CA supplementation tended to improve body weight (+ 15%, *P* = 0.09) and average daily gain (+ 21%, *P* = 0.07) of suckling piglets, along with increased levels of milk IgG (*P* = 0.01) and IgM (*P* = 0.02), colostrum crude fat (*P* = 0.01), and plasma glutathione peroxidase (GSH-Px) activity (*P* = 0.02) at farrowing. Moreover, maternal CA supplementation significantly improved plasma antioxidant capacity, expressions of intestinal barrier and anti-inflammatory genes, and gut microbiota structure of piglets at the end of suckling. Additionally, maternal CA supplementation increased the apparent total tract digestibility (ATTD) of crude protein (*P* < 0.01), gross energy (GE; *P* = 0.03), and dry matter (*P* = 0.01), improved jejunal sucrase activity (*P* < 0.01), villus height (*P* = 0.03), the ratio of villi height to crypt depth (*P* = 0.02), and the expressions of intestinal barrier and anti-inflammatory genes in post-weaning piglets. Furthermore, post-weaning CA supplementation tended to decrease diarrhea scores of piglets during d 14–21 and increased the ATTD of GE (*P* = 0.02), activities of jejunal sucrase (*P* = 0.02), plasma catalase (*P* = 0.01), and total superoxide dismutase (*P* < 0.01) in piglets.

**Conclusion:**

Maternal CA supplementation tended to increase the growth rate and weaning weight of suckling piglets, associated with improved antioxidant capacity and milk composition. Moreover, maternal CA supplementation or post-weaning CA supplementation improved nutrient digestibility, redox status, and intestinal function-related parameters of weaned piglets.

## Background

In modern intensive pig farming, high-yielding sows in late pregnancy and lactation experience severe oxidative stress due to metabolic burden [1]. The accumulation of reactive oxygen species (ROS) from the placenta and mammary gland in sows exceeds the elimination capacity of both enzymatic and non-enzymatic antioxidants, which negatively affects sow performance, milk production, and the growth rate of suckling piglets [2, 3]. Additionally, the gut microbiota is closely associated with oxidative stress, milk quality, and piglet health [4, 5]. Recently, maternal nutrition strategies have been employed to improve the performance and health of both sows and their offspring [6–8].

Weaning piglets face challenges such as maternal separation, dietary changes, and environmental shifts, which possibly lead to weaning stress [9]. This stress disrupts digestive and barrier function, induces oxidative stress and pro-inflammatory responses, and results in higher diarrhea incidence, lower feed intake, and growth rates in piglets [10–12].

Cinnamaldehyde (CA), an active component extracted from cinnamon, exhibits antioxidant, anti-inflammatory, and antimicrobial effects [13]. It has been reported that CA can inhibit inflammation by regulating the nuclear factor kappa B (NF-кB) pathway and reducing pro-inflammatory cytokines [14, 15]. Moreover, CA can enhance antioxidant capability and inhibit ROS production and oxidative stress [16, 17]. In animal models, dietary CA supplementation has improved inflammatory responses by regulating the NF-кB signaling pathway in fish [18], enhanced intestinal barrier in chickens [19], and benefited nursery pigs [20]. However, previous studies have primarily focused on the growth and health responses of piglets to CA supplementation. In this study, therefore, we investigated the effects of maternal and/or post-weaning dietary CA supplementation on sow reproductive performance and weaned piglet growth performance. Specifically, CA was added to the sow diet from d 107 of gestation to d 24 of lactation to investigate the effects of CA on reproductive performance, milk composition, and physiological traits of sows. Furthermore, split-pot experiments were conducted to investigate the effects of CA supplementation at different stages on growth performance, immunity, and gut health of weaned piglets.

## Materials and methods

### Experimental design

A total of 60 sows (parity 2 to 3) were randomly allocated into two groups at d 107 of gestation based on body condition score and parity. The sows in two groups were fed the CON diet (basal diet) or CA diet with basal diet supplemented with CA at 500 mg/kg (purity ≥ 45%, supplied by Yangzhidao Feed Co., Ltd., Jiangsu, China). The sow diets were fed from d 107 of gestation to d 24 of lactation. The basal diet of sows was formulated to meet or exceed the recommendations of Nutrient Requirements of Swine [21], as shown in Table 1.

**Table 1:** Ingredients and nutrient levels of the basal diet fed to sows (as-fed basis)

Ingredient	Content, %
Corn (CP 7.8%)	59.26
Dehulled soybean meal (CP 44.14%)	18.00
Fish meal (CP 62.5%)	2.00
Extruded soybean (CP 35.5%)	8.00
Sugar beet pulp	4.00
Wheat bran	4.00
Soybean oil	2.00
L-Lysine HCl	0.08
Choline chloride (50%)	0.20
Calcium carbonate	1.12
Calcium hydrogen phosphate	0.76
Sodium chloride	0.40
Mineral premix^a^	0.15
Vitamin premix^b^	0.03
Total	100.00
Nutrient level^c^	
Metabolizable energy, Mcal/kg	3.22
Crude protein	17.66
Crude fiber	3.76
Neutral detergent fiber	12.45
Calcium	0.78
Available phosphorus	0.27
SID lysine	0.85
SID methionine	0.26
SID threonine	0.54
SID tryptophan	0.17

^a^Mineral premix provided per kilogram of diet: Zn (ZnSO_4_·H_2_O), 100 mg; Cu (CuSO_4_·5H_2_O), 20 mg; Fe (FeSO_4_·H_2_O), 80 mg; Mn (MnSO_4_·H_2_O), 25 mg; I (KI), 0.14 mg; Se (Na_2_SeO_3_), 0.15 mg

^b^Vitamin premix provided per kilogram of diet: vitamin A, 7200 IU; vitamin D_3_, 1440 IU; vitamin E, 60 IU; vitamin K_3_, 2.88 mg; vitamin B_6_, 2.16 mg; vitamin B_1_, 1.20 mg; vitamin B_2_, 4.32 mg; biotin, 0.29 mg; folic acid, 2.4 mg; pantothenic acid, 15.00 mg

^c^Nutrient levels were calculated values

At the end of d 24 of lactation, a total of 128 piglets (initial body weight: 6.04 ± 0.10 kg) from 16 litters per treatment, according to body weight and source litter, were allotted into pens, and pens were assigned to dietary treatments in a split-plot design. Sows’ dietary treatment (CON diet or CA diet) served as the main plot and piglets’ dietary treatment (CON diet or CA diet) as a sub-plot. There were four groups with eight replicates and four piglets per replicate. The groups were as follows: 1) CON-CON (sows and piglets both fed CON diet); 2) CON-CA (sows fed CON diet and piglets fed CA diet); 3) CA-CON (sows fed CA diet and piglets fed CON diet); 4) CA-CA (sows and piglets both fed CA diet). The post-weaning experimental period lasted for 21 d, starting at weaning. The basal diet for piglets was formulated to meet or exceed the Nutrient Requirements of Swine [21], as presented in Table 2.

**Table 2:** Ingredients and nutrient levels of the basal diet fed to weaned piglets (as-fed basis)

Ingredient	Content, %
Corn (CP 7.8%)	24.80
Extruded corn (CP 8.70%)	25.00
Fermented soybean meal (CP 49.88%)	9.40
Enzymatic soybean (CP 38.5%)	5.00
Soybean concentrate protein (CP 65.20%)	5.00
Low-protein whey powder (CP 3.00%)	10.00
Whole milk powder (CP 23.60%)	8.00
Fish meal (CP 62.5%)	4.00
Coconut oil	1.00
Glucose	2.00
Sucrose	2.00
L-Lysine HCl	0.54
DL-Methionine	0.23
L-Threonine	0.23
L-Tryptophan	0.07
Choline chloride (50%)	0.16
Calcium carbonate	0.92
Calcium hydrogen phosphate	0.30
Sodium chloride	0.40
Zinc oxide (75%)	0.20
Acidifier	0.50
Mineral premix^a^	0.20
Vitamin premix^b^	0.05
Total	100.00
Nutrient level^c^	
Metabolizable energy, Mcal/kg	3.51
Calcium	0.83
Available phosphorus	0.43
SID lysine	1.43
SID methionine	0.53
SID threonine	0.84
SID tryptophan	0.26
SID valine	0.84
Lactose	10.96
Chemical analysis	
Gross energy, Mcal/kg	4.36
Crude protein	21.57
Ether extract	6.12

^a^Mineral premix provided per kilogram of diet: Zn (ZnSO_4_·H_2_O), 100 mg; Cu (CuSO_4_·5H_2_O), 100 mg; Fe (FeSO_4_·H_2_O), 100 mg; Mn (MnSO_4_·H_2_O), 4 mg; I (KI), 0.14 mg; Se (Na_2_SeO_3_), 0.30 mg

^b^Vitamin premix provided per kilogram of diet: vitamin A, 12,500 IU; vitamin D_3_, 2,500 IU; vitamin E, 40 IU; vitamin K_3_, 5.00 mg; vitamin B_6_, 6.00 mg; vitamin B_1_, 5.00 mg; vitamin B_2_, 12.50 mg; biotin, 0.25 mg; folic acid, 2.5 mg; pantothenic acid, 25.00 mg

^c^Nutrient levels were calculated values

### Feed management

On d 107 of gestation, sows were moved to farrowing crates (2.13 m × 0.66 m) in a farrowing room with environmental control systems (maintained at 24–26 °C). From d 107 to d 111 of gestation, sows were fed 3.2 kg of diet daily, split into two equal meals at 08:00 and 16:00. From d 112 of gestation until farrowing, the daily feed intake was reduced to 2.0 kg, also provided in two equal meals at the same times. On the day of parturition, sows were not fed; they received 2 kg the next day, with a gradual increase of 0.5 kg/d until ad libitum. Throughout the experimental period, CA was evenly mixed into the basal diet and manually administered to the sows’ troughs. All sows and their suckling piglets had free access to water. At the time of parturition, data on the number of total piglets born, stillborn piglets, mummified piglets, piglet weights, and farrowing duration were recorded. Within 48 h after birth, litter size was standardized to be 12 ± 1 piglets per sow through cross-fostering within the same group. All piglets were weighed at 48 h after birth and weekly during lactation to calculate average daily gain (ADG). During lactation, sow milk was the sole nutrient source for the suckling piglets. Meanwhile, feed intake of sows was recorded during lactation to calculate the average daily feed intake (ADFI).

On the morning of d 25 of lactation, a total of 128 suckling piglets were transferred to the nursery room. All weaned piglets had free access to water and were fed ad libitum in this period. Meanwhile, the body weight and feed intake of the weaned piglets were recorded weekly to calculate ADG, ADFI, and the ratio of ADFI to ADG (F:G). Diarrhea scores were monitored three times daily (at 08:00, 12:00, and 16:00). Fecal scores were determined based on the appearance of feces on the floor using a 4-point scale: 0 = solid and well-formed, 1 = soft and formed feces, 2 = fluid and yellowish feces, and 3 = watery and projectile feces. Diarrhea scores were calculated using the equation: diarrhea scores = the sum of diarrhea scores (numbers of piglets per pen × experimental days × assessed times per day).

### Sample collection

Eight to ten sows were randomly selected from each group for sample collection. Blood samples (10 mL) were collected from the ear vein of ten sows in each group at 1 h after the onset of farrowing and on d 25 of lactation. The samples were then centrifuged at 3,000 × *g* for 15 min to separate plasma. Colostrum samples (20 mL) were manually collected from all functional teats of the sows within 1 h after the onset of farrowing. On d 8 of lactation, milk samples (20 mL) were collected by infusing the sows with 5 mL of oxytocin. Colostrum and milk samples were centrifuged at 12,000 × *g* for 1 h at 4 °C to obtain whey samples for further analysis. Fecal samples (2 g) were freshly collected from eight sows per group by rectal grab on d 25 of lactation. All plasma, colostrum, milk, colostrum whey, milk whey, and fecal samples were stored at −80 °C for further analysis. Eight piglets per treatment group at postnatal day 24 (PND 24) with body weight closest to the average were selected for plasma collection and then euthanized. The middle 4-cm segments of the ileum were dissected and rinsed in PBS, and the mucosa was scraped off with a glass slide before being stored in liquid nitrogen. Colonic contents were collected from the mid-colon segment of each piglet and stored at −80 °C for further analysis.

Chromic oxide, an indigestible marker, was added to diets at 0.3% for the digestion test. From d 12 to 14 after weaning, fresh fecal samples and diets (200 g each) were collected, then dried at 65 °C for 72 h, ground through a 0.42-mm screen, and stored at −20 °C for further analysis. Weaned piglets at 45 days of age were individually weighed, and those closest to the average weight (10.63 ± 0.12 kg) per pen were selected for plasma samples (*n* = 8 piglets/treatment) and then euthanized. About 5 cm segments of mid-jejunum were fixed with 4% paraformaldehyde solution for morphological analysis, while the remaining jejunal mucosa samples were scraped and collected using a sterile glass microscope slide. Ileal tissue samples were flushed with ice-cold saline and collected into liquid nitrogen. All samples were stored at −80 °C for further analysis.

### Chemical analysis

The diets and fecal samples were analyzed for crude protein (CP), dry matter (DM), and ether extract (EE) following the methods of the Association of Official Agricultural Chemists [22]. The gross energy (GE) of diets and feces was determined using an automatic adiabatic oxygen bomb calorimeter (Parr Instrument Co., Moline, IL, USA). The Cr_2_O_3_ content was measured by a flame atomic absorption spectrophotometer (ContrAA 700, Analytikjena, Jena, Germany).

### Milk composition

The concentrations of fat, protein, lactose, DM, and urea nitrogen in colostrum and milk were analyzed by an automatic milk composition analyzer (MilkoScan™ FT+, FOSS, Hilleroed, Denmark). The levels of immunoglobulin G (IgG) and immunoglobulin M (IgM) in colostrum whey and milk whey were determined using an automatic biochemical analyzer (Hitachi 3100, Tokyo, Japan) with corresponding commercial kits (Sichuan Maccura Biotechnology Inc., Chengdu, China).

### Plasma parameters

The concentrations of glucose (catalog No. CH0101102), total cholesterol (TG, catalog No. CH0101151), urea (catalog No. CH0101051), non-esterified fatty acid (NEFA, catalog No. RB30951), total bile acid (TBA, catalog No. RB11301), C-reactive protein (CRP, catalog No. CH0105303), IgG (catalog No. IGM0011), IgM(catalog No. IGG0011), alanine aminotransferase (ALT, catalog No. CH0101201), aspartate amino transferase (AST, catalog No. CH0101202), gamma-glutamyl transpeptidase (GGT, catalog No. CH0101204), and complement C3 (C3, catalog No. C300011) in plasma were determined by using an automatic biochemical analyzer (Hitachi 3100, Tokyo, Japan) through corresponding commercial kits (Sichuan Maccura Biotechnology Inc., Chengdu, China).

The activities of total superoxide dismutase (T-SOD, catalog No. A001-1-2), catalase (CAT, catalog No. A007-1-1), glutathione peroxidase (GSH-Px, catalog No. A005-1-2), and the content of malondialdehyde (MDA, catalog No. A003-1-2) in plasma were measured using commercially available kits (Nanjing Jiancheng Bioengineering Institute, Nanjing, China) following the manufacturer’s instruction.

The concentrations of IL-1β (catalog No. H002-1-2), interleukin-10 (IL-10, catalog No. A009- 1-2), and TNF-α (catalog No. H052-1-2) were determined by ELISA kits (Nanjing Jiancheng Bioengineering Institute, Nanjing, China) according to the manufacturer’s instruction.

### Jejunal morphology

The jejunal samples from weaned piglets were taken out of the fixative solution, dehydrated, and embedded in paraffin. Each jejunal sample was sectioned at 5-μm thickness and stained with hematoxylin and eosin. Ten representative villi from each section were used to measure the villus height and crypt depth by magnification (Nikon, Tokyo, Japan). The ratio of villus height to crypt depth (VCR) was calculated.

### Digestive enzyme analysis

The jejunal mucosa samples of weaned piglets were homogenized in ice-cold saline at the ratio of 1:9 (g/mL) and then centrifuged at 3,000 × *g* for 10 min at 4 °C to collect supernatant. The activities of maltase, sucrase, aminopeptidases A (APA), and aminopeptidases N (APN) were determined by commercial kits (Nanjing Jiancheng Bioengineering Institute, Nanjing, China) according to the manufacturer’s instructions.

### RNA extraction and quantitative real-time PCR analysis

Total RNA was extracted from 0.1 g of ileal tissue of piglets at PND 24 and post-weaned day 21 (PWD 21) samples using TRIZOL reagent (TaKaRa Biotechnology, Dalian, China) following the manufacturer’s protocol. The concentration and purity of extracted RNA were determined by using a nucleic acid analyzer (Beckman DU-800; Beckman Coulter, Inc., Brea, CA, USA). Then, total RNA was reverse transcribed into cDNA using the PrimeScripte RT reagent kit (TaKaRa Biotechnology, Dalian, China) according to the manufacturer’s instructions. All real-time PCR was performed using SYBR Premix Ex Taq reagents (TaKaRa Biotechnology, Dalian, China) on a CFX96 Real-Time PCR System (Bio-Rad, Hercules, CA, USA). β-actin served as a housekeeping gene to normalize the mRNA expression levels of the target genes according to the 2^−ΔΔCt^ method [23], and the primers were presented in Table 3.

**Table 3:** Primer sequences used for quantitative real-time polymerase chain reaction

Gene	Primer sequence (5′→3′)	GeneBank accession
*TLR-4*	F: AGAAAATATGGCAGAGGTGAAAGC	GQ304754
	R: CTTCGTCCTGGCTGGAGTAGA	
*TLR-9*	F: AATCCAGTCGGAGATGTTTGCT	AY859728
	R: GACCGCCTGGGAGATGCT	
*MyD88*	F: GTGCCGTCGGATGGTAGTG	NM_001099923
	R: TCTGGAAGTCACATTCCTTGCTT	
*NF-κB*	F: TGCTGGACCCAAGGACATG	AK348766.1
	R: CTCCCTTCTGCAACAACACGTA	
*CLDN1*	F: GATCGGCTCCATCGTCAGCA	NM_001244539.1
	R: CATTGACTGGGGTCATGGGGTC	
*OCLN*	F: TTCATTGCTGCATTGGTGAT	NM_0011636471
	R: ACCATCACACCCAGGATAGC	
*ZO-1*	F: CCGCCTCCTGAGTTTGATAG	AJ318101
	R: CAGCTTTAGGCACTGTGCTG	
*SOD1*	F: GAGCTGAAGGGAGAGAAGACAGT	NM_001190422.1
	R: GCACTGGTACAGCCTTGTGTAT	
*SOD2*	F: CTGGACAAATCTGAGCCCTAAC	NM_214127.2
	R: GACGGATACAGCGGTCAACT	
*GPX1*	F: CACAACGGTGCGGGACTA	NM_214201.1
	R: CATTGCGACACACTGGAGAC	
*CAT*	F: CGAAGGCGAAGGTGTTTG	NM_214301.2
	R: AGTGTGCGATCCATATCC	
*TNF-α*	F: CCACGTTGTAGCCAATGTCA	X57321
	R: CAGCAAAGTCCAGATAGTCG	
*IL-1β*	F: TCTGCCCTGTACCCCAACTG	NM214055.1
	R: CCAGGAAGACGGGCTTTTG	
*IL-6*	F: GATGCTTCCAATCTGGGTTCA	M80258.1
	R: CACAAGACCGGTGGTGATTCT	
*IL-10*	F: CACGGCCTTGCTCTTGTTTT	NM_214041.1

*TLR-4* Toll-like receptor 4, *TLR-9* Toll-like receptor 9, *Myd88* Myeloid differentiation factor 88, *NF-κB* Nuclear factor kappa B, *CLDN1 *Claudin 1, *OCLN* Occludin, *ZO-1* Zonula occludens-1, *SOD1* Superoxide dismutase 1, *SOD2* Superoxide dismutase 2,* GPX1* Glutathione peroxidase 1, *CAT* Catalase, *TNF-α* Tumor necrosis factor-α, *IL-1β* Interleukin 1-β, *IL-6* Interleukin 6, *IL-10* Interleukin 10

### Sequencing of gut microbiota

Microbial genomic DNA was extracted from the feces of sows and colonic digesta of piglets at PND 24 using the CATB method. 1% (w/v) agarose gel electrophoresis was used to detect the integrity of extracted genomic DNA. The v4 hypervariable regions of 16S rRNA were amplified using primers 515F (5′-GTGYCAGCMGCCGCGGTAA-3′) and 806R (5′-GGACTACHVGGGTWTCTAAT-3′). The library was constructed by the NEB Next Ultra DNA library prep kit (Illumina, USA). Then, the library was sequenced on an Illumina HiSeq platform. Operational taxonomic units were generated based on the 97% sequence identity. The α-diversity was calculated from this output normalized data, and β-diversity was performed by principal coordinate analysis (PCoA).

### Short-chain fatty acid analysis

The concentration of short-chain fatty acids (SCFAs) in sow fecal samples was determined using a CP-3800 gas chromatography instrument (Varian Medical Systems, Palo Alto, CA, USA). Specifically, the mixture of 0.5 g of colonic digesta samples with 1.5 mL of water was allowed to stand for 30 min and then centrifuged at 10,000 × *g* for 30 min at 4 °C to collect supernatant. 1 mL of supernatant was mixed with 0.2 mL of metaphosphoric acid and 23.3 μL of crotonic acid and centrifuged at 8,000 × *g* for 10 min. Then, 0.3 mL of supernatant was mixed with 0.9 mL of chromatographic methanol, centrifuged at 8,000 × *g* for 5 min, and filtered by a 0.22-μm filter membrane for analysis.

### Statistical analysis

All data were checked for variance homogeneity and normality using the Shapiro–Wilk test and Levene’s test procedures of SAS software (Version 9.4, SAS Inst. Inc., Cary, NC, USA), respectively. Each sow served as an experimental unit, and suckling piglet was reported as a mean for the litter. The data were analyzed by an independent *t*-test of SAS. In the post-weaning study, the MIXED procedure in SAS for a split-plot arrangement with sow diet as the whole plot and piglet diet as the split plot was used to analyze data on growth performance, apparent total tract digestibility (ATTD), diarrhea scores, plasma biochemical parameters, gut morphology, barrier function, and microbiota data. Results were presented as means with pooled SEM. A *P*-value of less than 0.05 was considered significant, while a *P*-value between 0.05 and 0.10 was indicated as a tendency.

## Results

### Reproductive performance

Maternal CA supplementation had no significant effects on litter performance at parturition or ADFI of sows during lactation. However, maternal CA supplementation tended to increase ADG during lactation (*P* = 0.07) and the body weight (*P* = 0.09) of piglets at the end of suckling compared with the CON group (Table 4).

**Table 4:** Effects of maternal CA supplementation on sow performance

Item	CON	CA	SEM	*P*-value
**At farrowing**				
Total pigs born, n	15.82	15.76	1.08	0.95
Pigs born alive, n	14.57	14.84	1.00	0.79
Stillborn, n	0.89	0.72	0.30	0.57
Mummy, n	0.36	0.20	0.16	0.31
LBW piglets, n	0.39	0.24	0.20	0.45
Total born, kg				
Litter birth weight	19.42	20.51	1.27	0.39
Piglet birth weight	1.29	1.33	0.06	0.51
Born alive, kg				
Litter birth weight	18.52	19.64	1.26	0.37
Piglet birth weight	1.30	1.33	0.06	0.57
Farrowing duration, min	186	165	17	0.24
Mean birth interval, min	12.33	10.94	1.27	0.28
**At lactation**				
Litter size, n				
Cross-foster	12.50	12.43	0.26	0.78
Piglets weaned	11.67	11.79	0.28	0.67
Piglet weight, kg				
Cross-foster	1.87	1.87	0.16	0.98
Piglets weaned	5.78	6.66	0.50	0.09
ADG of piglets during lactation, g/d	193	234	22	0.07
ADFI of sows during lactation, kg/d	6.48	6.38	0.17	0.56

*CON* Basal diet, *CA* Basal diet + 500 mg/kg cinnamaldehyde, *LBW* Piglets with birth weight less than 0.70 kg, *ADG* Average daily gain, *ADFI* Average daily feed intake

### Colostrum and milk composition

Maternal CA supplementation increased (*P* < 0.05) fat content in colostrum, IgG, and IgM in the milk compared with the CON group (Table 5).

**Table 5:** Effects of maternal CA supplementation on colostrum and milk

Item	CON	CA	SEM	*P*-value
Colostrum				
Crude fat, %	4.49	5.40	0.35	0.01
Protein, %	16.48	16.69	0.83	0.79
Lactose, %	2.90	2.93	0.17	0.85
Dry matter, %	28.35	29.06	0.94	0.46
Urea nitrogen, mg/dL	65.64	67.00	4.82	0.78
IgG, g/L	3.96	3.98	0.20	0.93
IgM, g/L	0.76	0.85	0.10	0.38
Milk				
Crude fat, %	10.38	11.19	1.17	0.50
Protein, %	7.46	7.57	0.29	0.70
Lactose, %	7.61	7.72	0.20	0.58
Dry matter, %	28.27	29.17	1.38	0.53
Urea nitrogen, mg/dL	84.53	78.00	4.88	0.21
IgG, g/L	0.72	0.93	0.07	0.01
IgM, g/L	0.29	0.51	0.07	0.02

*CON* Basal diet, *CA* Basal diet + 500 mg/kg cinnamaldehyde, *IgG* Immunoglobulin G, *IgM* Immunoglobulin M

### Parameters related to metabolism and redox status of sows and offspring

Maternal CA supplementation increased the activity of plasma GSH-Px and tended to increase the plasma glucose concentration (*P* = 0.06) of sows at farrowing. In addition, maternal CA supplementation decreased the concentration of plasma TBA (*P* < 0.05) and tended to increase the activity of T-SOD (*P* = 0.05) of sows at the end of lactation, as compared with the CON group. Likewise, maternal CA supplementation increased (*P* < 0.05) the plasma activities of GSH-Px and CAT in piglets at PND 24, as compared with the CON group (Table 6).

**Table 6:** Effects of maternal CA supplementation on plasma parameters related to metabolism and redox status of sows and piglets at PND 24

Item	CON	CA	SEM	*P*-value
**Sow**				
At farrowing				
Glucose, mmol/L	5.19	5.58	0.20	0.06
TG, mmol/L	0.26	0.25	0.03	0.70
UREA, mmol/L	4.23	4.15	0.21	0.69
NEFA, μmol/L	954.28	913.56	105.58	0.70
TBA, μmol/L	16.67	11.29	3.38	0.12
ALT, U/L	39.11	41.38	2.96	0.45
AST, U/L	37.02	36.57	4.42	0.92
GGT, U/L	35.11	32.10	3.21	0.36
GSH-Px, U/mL	4,191.25	4,278.87	34.19	0.02
MDA, nmol/mL	1.24	1.20	0.20	0.84
CAT, U/mL	5.94	6.56	0.65	0.35
T-SOD, U/mL	174.32	179.68	6.05	0.38
At the end of lactation				
Glucose, mmol/L	5.07	5.39	0.57	0.59
TG, mmol/L	0.31	0.27	0.09	0.66
UREA, mmol/L	5.39	5.73	0.86	0.69
NEFA, μmol/L	91.69	94.63	24.07	0.90
TBA, μmol/L	39.78	16.99	7.09	< 0.01
ALT, U/L	38.46	40.80	3.68	0.53
AST, U/L	24.8	26.05	3.01	0.68
GGT, U/L	38.44	44.06	5.88	0.35
GSH-Px, U/mL	3,889.57	3,888.37	39.83	0.97
MDA, nmol/mL	1.49	1.27	0.28	0.42
CAT, U/mL	4.52	5.14	0.84	0.47
T-SOD, U/mL	144.65	154.79	4.91	0.05
**Piglets at PND 24**				
GSH-Px, U/mL	2,169.32	2,498.37	125.29	0.01
MDA, nmol/mL	2.66	1.87	0.53	0.15
CAT, U/mL	6.17	11.08	1.96	0.02
T-SOD, U/mL	44.27	46.70	2.21	0.28

*CON* Basal diet, *CA* Basal diet + 500 mg/kg cinnamaldehyde, *TG* Triglyceride, *NEFA* Non-esterified fatty acid, *TBA* Total bile acid, *ALT* Alanine aminotransferase, *AST* Aspartate aminotransferase, *GGT* Gamma-glutamyl transpeptidase, *GSH-Px* Glutathione peroxidase, *MDA* Malondialdehyde, *CAT* Catalase, *T-SOD* Total superoxide dismutase, *PND* Postnatal day

### Inflammatory cytokines and immunoglobulins of sows and offspring

Maternal CA supplementation had no significant effects on the plasma concentrations of TNF-α, IL-1β, IL-10, CRP, IgG, and IgM of sows at farrowing or end of lactation, as compared with the CON group (Table 7). However, maternal CA supplementation tended to increase plasma IgM concentration (*P* = 0.05) in piglets at PND 24, as compared with the CON group (Table 7).

**Table 7:** Effects of maternal CA supplementation on immunological and inflammatory parameters in plasma of sows and piglets at PND 24

Item	CON	CA	SEM	*P*-value
**Sow**				
At farrowing				
TNF-α, ng/L	458.77	422.55	42.07	0.40
IL-1β, ng/L	14.30	12.85	2.94	0.62
IL-10, ng/L	209.96	223.75	22.23	0.54
CRP, mg/L	7.35	6.58	0.46	0.11
IgG, g/L	3.86	3.88	0.10	0.83
IgM, g/L	1.12	1.20	0.05	0.11
At the end of lactation				
TNF-α, ng/L	483.98	466.61	22.32	0.46
IL-1β, ng/L	19.70	15.06	2.80	0.11
IL-10, ng/L	268.47	251.75	23.01	0.49
CRP, mg/L	10.90	10.48	1.07	0.70
IgG, g/L	4.25	4.31	0.09	0.45
IgM, g/L	0.76	0.85	0.12	0.45
**Piglets at PND 24**				
TNF-α, ng/L	237.14	168.70	48.11	0.18
IL-1β, ng/L	22.66	21.83	2.70	0.76
C3, g/L	0.05	0.06	0.01	0.26
IgG, g/L	3.40	3.62	0.24	0.35
IgM, g/L	0.20	0.26	0.03	0.05

*CON* Basal diet, *CA* Basal diet + 500 mg/kg cinnamaldehyde, *TNF-α* Tumor necrosis factor-α, *IL-1β* Interleukin 1-β, *IL-10* Interleukin 10, *CRP* C-reactive protein*, IgG* Immunoglobulin G, *IgM* Immunoglobulin M, *PND* Postnatal day, *C3* Complement C3

### Gene expressions of piglets at PND 24

**Fig. 1:**
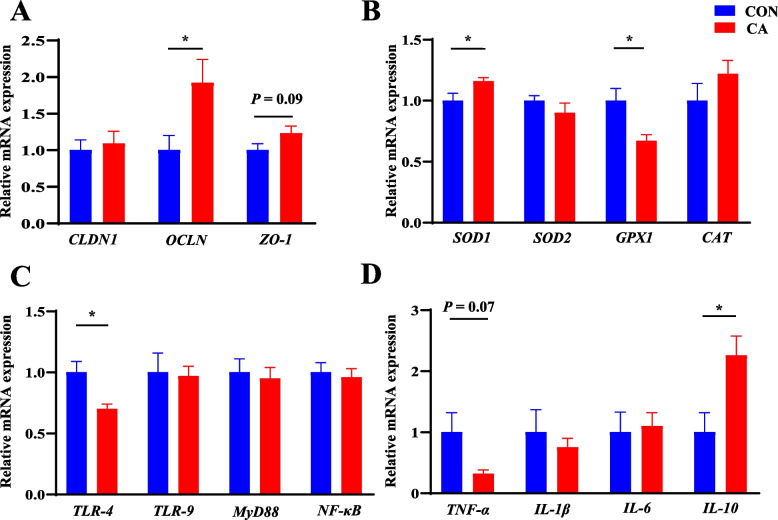
Effects of maternal CA supplementation on expressions of genes related to intestinal barrier, redox status and immunity of piglets at PND 24. **A** mRNA expressions of intestinal barrier-related genes in ileal tissue. **B** mRNA expressions of redox status-related genes in ileal tissue. **C** mRNA expressions of innate immunity-related genes in ileal tissue. **D** mRNA expressions of inflammatory genes in ileal tissue. *CLDN1* Claudin 1, *OCLN* Occludin, *TLR-4* Toll-like receptor 4, *TLR-9* Toll-like receptor 9, *Myd88* Myeloid differentiation factor 88, *NF-κB* Nuclear factor kappa B, *ZO-1* Zonula occludens-1, *SOD1* Superoxide dismutase 1, *SOD2* Superoxide dismutase 2,* GPX1* Glutathione peroxidase 1, *CAT* Catalase, *TNF-α* Tumor necrosis factor-α, *IL-1β* Interleukin 1-β, *IL-6* Interleukin 6, *IL-10* interleukin 10. *CON* Basal diet, *CA* Basal diet + 500 mg/kg cinnamaldehyde. Data expressed as mean ± a pooled SEM. ^*^P < 0.05

Maternal CA supplementation increased mRNA expressions of *OCLN* (*P* < 0.05), *ZO-1* (*P* = 0.09), *SOD1* (*P* < 0.05), and *IL-10* (*P* < 0.05) in the ileum, whereas *GPX1* (*P* < 0.05), *TLR-4* (*P* < 0.05), and *TNF-α* (*P* = 0.07) mRNA expressions in the ileum were decreased in piglets at PND 24 compared with the CON group (Fig. 1).

### Gut microbiota and fecal short-chain fatty acids of sows and offspring

**Fig. 2:**
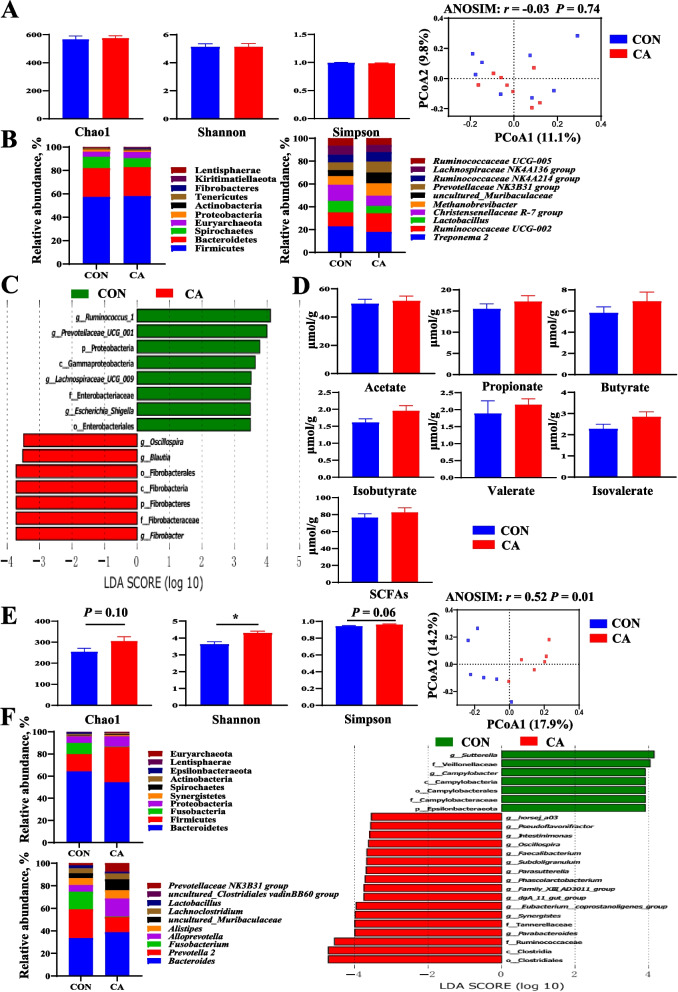
Effects of maternal CA supplementation on gut microbiota and short chain fatty acid of sows and piglets at PND 24. **A** Alpha diversity index and principal coordinate analysis of sows. **B** Predominant microorganisms at phylum and genus level of sows. **C** LEfSe analysis of sows. **D** Concentrations of fecal SCFAs of sows. **E** Alpha diversity index and principal coordinate analysis of piglets at PND 24. **F** Predominant microorganisms at phylum and genus level, as well as LEfSe analysis of piglets at PND 24. *CON* Basal diet, *CA* Basal diet + 500 mg/kg cinnamaldehyde. Data expressed as mean ± a pooled SEM

There were no significant differences in Chao 1, Shannon, Simpson index, and PCoA of sows between CON and CA groups (Fig. 2A). At the phylum level, maternal CA supplementation increased the fecal Fibrobacteres abundance but decreased the Proteobacteria abundance of sows at the end of lactation (Fig. 2B). At the genus level, maternal CA supplementation decreased *Christensenellaceae R-7 group* abundance, but increased *uncultured_Muribaculaceae* abundance of sows at the end of lactation (Fig. 2B). LEfSe analysis showed that the CA group had higher relative abundances of *g_Oscillospira*, *g_Blautia*, and o_Fibrobacterales, while the CON group had higher relative abundances of *g_Ruminococcus_1*, *g_Prevotellacease_UCG_001*, p_Proteobacteria, c_Gammaproteobactria, *g_Lachnospiraceae_UCG_009*, and f_Enterobacteiaceae (Fig. 2C). In addition, there were no significant differences in fecal concentrations of acetate, propionate, butyrate, isobutyrate, isovalerate, valerate, and total SCFA between CON and CA groups (Fig. 2D).

Maternal CA supplementation increased the Shannon index (*P* < 0.05) and tended to increase Chao1 (*P* = 0.10) and Simpson (*P* = 0.06) index in piglets at PND 24 compared with the CON group (Fig. 2E). Meanwhile, there were significant differences in the colonic microbial composition of piglets at PND 24 from sows fed a CON diet or CA diet in beta diversity, as shown by PCoA (Fig. 2E). At the phylum level, maternal CA supplementation increased abundances of Firmicutes, Lentisphaerae, and Proteobacteria and decreased abundances of Epsilonbacteraeota and Fusobacteria in piglets at PND 24 (Fig. 2F). At the genus level, maternal CA supplementation decreased the abundances of *Fusobacterium* and *Prevotella 2* in piglets at PND 24 (Fig. 2F). LEfSe analysis showed that piglets at PND 24 from sows fed the CON diet had higher relative abundances of *g_Sutterella*, f_Veillonellaceae, *g_Campylobacter*, and p_Epsilonbacteraeota, while piglets from sows fed the CA diet had higher relative abundances of *g_horsej_a03*, *g_Pseudoflavonifractor*, *g_Intestinimonas*, *g_Oscillospira*, *g_Faecalibacterium*, *g_Subdoligranulum*, *g_Parasutterella*, *g_Phascolarctobacterium*, *g_Family_XIII_AD3011_group*, *g_daA_11_gut_group*, *g_Eubacterium_coprostanoligenes_group*, *g_Synergistes*, f_Tannerellaceae, *g_Parabacteroides*, *f_ Ruminococcaceae*, and c_Clostridia (Fig. 2F).

### Growth performance and diarrhea scores of weaned piglets

Diarrhea scores (*P* = 0.06) and F:G (*P* < 0.05) in weaned piglets from CA-fed sows were lower than those in piglets from CON-fed sows during d 8–14. Meanwhile, diarrhea scores in weaned piglets with post-weaning CA supplementation were lower than those in post-weaning CON piglets (*P* < 0.05) during d 15–21 (Table 8).

**Table 8:** Effects of dietary CA supplementation in sows or piglets on growth performance of weaned piglets

Sow diet	CON		CA		SEM	*P*-value		
**Piglet diet**	**CON**	**CA**	**CON**	**CA**		**Sow**	**Piglet**	**Sow × Piglet**
Body weight, kg								
1 d	6.10	6.03	6.01	6.01	0.12	0.82	0.89	0.88
8 d	6.95	6.83	6.77	6.78	0.12	0.61	0.87	0.78
15 d	8.17	8.11	8.09	8.16	0.13	0.95	0.99	0.81
22 d	10.68	10.73	10.52	10.59	0.12	0.55	0.82	0.97
ADG, g/d								
1–7 d	120	115	105	109	5	0.32	0.94	0.66
8–14 d	175	182	193	197	10	0.36	0.76	0.94
15–21 d	359	376	346	347	12	0.34	0.67	0.70
ADFI, g/d								
1–7 d	220	196	189	182	8	0.16	0.34	0.60
8–14 d	383	362	339	344	10	0.13	0.68	0.52
15–21 d	592	575	566	537	14	0.27	0.43	0.84
F:G								
1–7 d	1.87	1.76	1.80	1.69	0.08	0.71	0.57	0.99
8–14 d	2.26	1.94	1.77	1.69	0.09	0.03	0.24	0.49
15–21 d	1.67	1.54	1.64	1.53	0.03	0.84	0.13	0.89
Diarrhea scores								
1–7 d	0.85	1.05	1.08	0.96	0.08	0.65	0.81	0.33
8–14 d	0.77	0.55	0.50	0.46	0.05	0.06	0.16	0.35
15–21 d	0.46	0.34	0.39	0.31	0.03	0.37	0.07	0.73

*CON* Basal diet, *CA* Basal diet + 500 mg/kg cinnamaldehyde, *ADG* Average daily gain, *ADFI* Average daily feed intake, *F:G* The radio of ADFI to ADG

### ATTD of nutrients of weaned piglets

The ATTD of CP, GE, and DM in weaned piglets from CA-fed sows was higher than that in piglets from CON-fed sows (*P* < 0.05, Table 9). Meanwhile, the ATTD of GE in weaned piglets with post-weaning CA supplementation was higher than that in post-weaning CON piglets (*P* < 0.05).

**Table 9:** Effects of dietary CA supplementation in sows or piglets on apparent total tract digestibility of weaned piglets

Sow diet	CON		CA		SEM	*P*-value		
**Piglet diet**	**CON**	**CA**	**CON**	**CA**		**Sow**	**Piglet**	**Sow × Piglet**
CP, %	65.98	69.67	73.86	73.63	0.08	< 0.01	0.24	0.18
EE, %	81.31	81.49	84.66	85.35	0.05	0.06	0.81	0.89
GE, %	77.78	81.50	81.33	82.74	0.03	0.03	0.02	0.29
DM, %	79.82	79.78	82.94	82.36	0.03	0.01	0.77	0.79

*CON* Basal diet, *CA* Basal diet + 500 mg/kg cinnamaldehyde, *CP* Crude protein, *EE* Ether extract, *GE* Gross energy, *DM* Dry matter

### Activities of digestive enzymes and intestinal morphology of weaned piglets

**Fig. 3:**
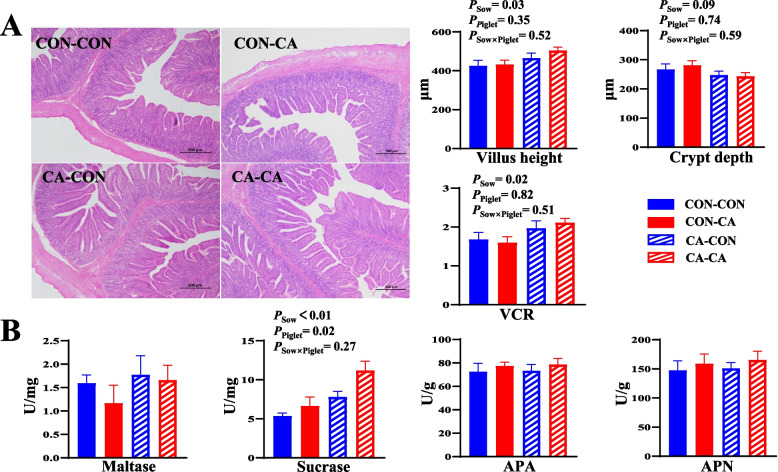
Effects of dietary CA supplementation in sows or piglets on intestinal morphology and activities of digestive enzymes of weaned piglets. **A** Intestinal morphology of jejunal morphology. **B** Activities of digestive enzymes. *CON-CON* Sows and piglets both fed basal diet, *CON-CA* Sows fed basal diet and piglets fed basal diet + 500 mg/kg cinnamaldehyde, *CA-CON* Sows fed basal diet + 500 mg/kg cinnamaldehyde and piglets fed basal diet*, CA-CA* Sows and piglets both fed basal diet + 500 mg/kg cinnamaldehyde, *VCR* The ratio of villi height-to-crypt depth. Data expressed as mean ± a pooled SEM

The villus height, VCR, and sucrase activity of the jejunum in weaned piglets from CA-fed sows were higher than those in piglets from CON-fed sows (*P* < 0.05, Fig. 3A and B). Meanwhile, the sucrase activity of the jejunum in weaned piglets with post-weaning CA supplementation was higher than that in post-weaning CON piglets (*P* < 0.05, Fig. 3B).

### Antioxidant capacity, inflammatory cytokines and immunoglobulins of weaned piglets

**Fig. 4:**
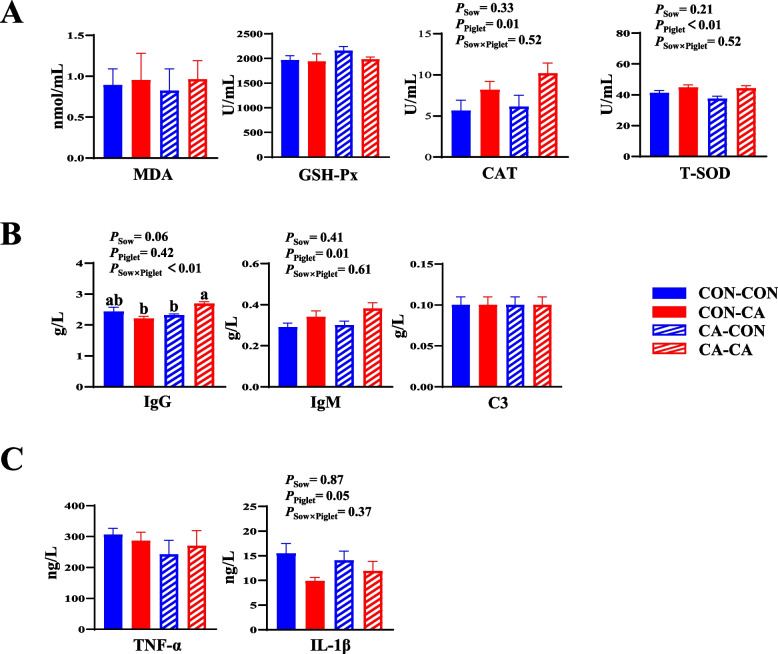
Effects of dietary CA supplementation in sows or piglets on redox status, immunological and inflammatory response of weaned piglets. **A** Plasma redox status-related parameters of weaned piglets. **B** Plasma immunological parameters of weaned piglets. **C** Plasma inflammatory cytokine of weaned piglets. *GSH-Px* Glutathione peroxidase, *MDA* Malondialdehyde, *CAT* Catalase, *T-SOD* Total superoxide dismutase, *TNF-α* Tumor necrosis factor-α, *IL-1β* Interleukin 1-β, *IgG* Immunoglobulin G, *IgM* Immunoglobulin M, *C3* Complement C3. *CON-CON* Sows and piglets both fed basal diet, *CON-CA* Sows fed basal diet and piglets fed basal diet + 500 mg/kg cinnamaldehyde, *CA-CON* Sows fed basal diet + 500 mg/kg cinnamaldehyde and piglets fed basal diet*, CA-CA* Sows and piglets both fed basal diet + 500 mg/kg cinnamaldehyde. Data expressed as mean ± a pooled SEM. ^a, b^Mean values with different superscripts letters are significantly different (*P* < 0.05)

The plasma activities of T-SOD and CAT and the IgM plasma concentration in weaned piglets with post-weaning CA supplementation were higher than those in post-weaning CON piglets (*P* < 0.05, Fig. 4A and B). There was an interaction between the CA-supplemented diet for sows and the CA-supplemented diet for weaned piglets on increasing the plasma concentration of IgG (*P* < 0.05, Fig. 4B). Weaned piglets from the CA-CA group had higher IgG concentration in plasma compared with weaned piglets from the CON-CA or CA-CON group (*P* < 0.05, Fig. 4B).

### Gene expressions

**Fig. 5:**
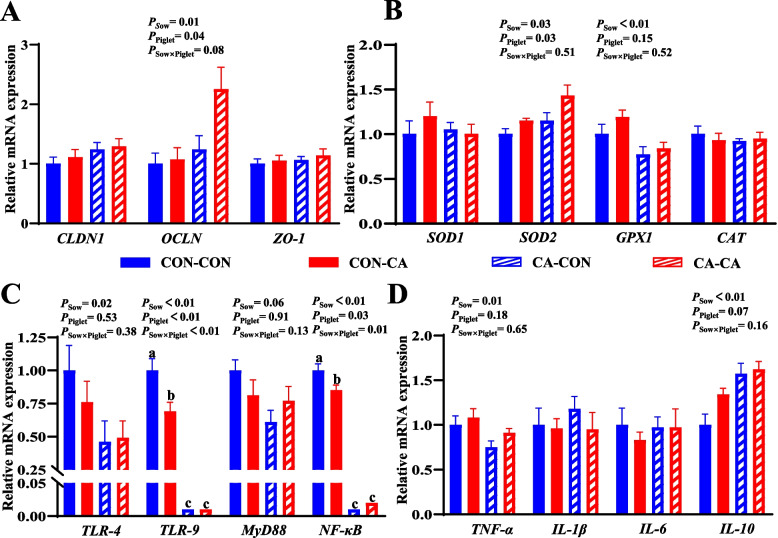
Effects of dietary CA supplementation in sows or piglets on expressions of genes related to intestinal barrier, redox status and immunity of weaned piglets. **A** mRNA expressions of intestinal barrier-related genes in ileal tissue. **B** mRNA expressions of redox status-related genes in ileal tissue. **C** mRNA expressions of innate immunity-related genes in ileal tissue. **D** mRNA expressions of inflammatory genes in ileal tissue. *CLDN1* Claudin 1, *OCLN* Occludin, *TLR-4* Toll-like receptor 4, *TLR-9* Toll-like receptor 9, *Myd88* Myeloid differentiation factor 88, *NF-κB* Nuclear factor kappa B, *ZO-1* Zonula occludens-1, *SOD1* Superoxide dismutase 1, *SOD2* Superoxide dismutase 2,* GPX1* Glutathione peroxidase 1, *CAT* Catalase, *TNF-α* Tumor necrosis factor-α, *IL-1β* Interleukin 1-β, *IL-6* Interleukin 6, *IL-10* Interleukin 10. *CON-CON* Sows and piglets both fed basal diet, *CON-CA* Sows fed basal diet and piglets fed basal diet + 500 mg/kg cinnamaldehyde, *CA-CON* Sows fed basal diet + 500 mg/kg cinnamaldehyde and piglets fed basal diet*, CA-CA* Sows and piglets both fed basal diet + 500 mg/kg cinnamaldehyde. Data expressed as mean ± a pooled SEM. Mean values with different superscripts letters are significantly different (*P* < 0.05)

Weaned piglets from CA-fed sows had higher mRNA expressions of *SOD2* and *IL-10* (*P* < 0.05) in the ileum and lower mRNA expressions of *GPX1* (*P* < 0.05), *TLR-4* (*P* < 0.05), and *MyD88* (*P* = 0.06) in the ileum than those in weaned piglets from CON-fed sows (Fig. 5B–D). Meanwhile, ileum mRNA expressions of *SOD2* (*P* < 0.05) and *IL-10* (*P* = 0.07) in weaned piglets with post-weaning CA supplementation were higher than those in post-weaning CON piglets (Fig. 5B and D). There was an interaction between the CA-supplemented diet for sows and the CA-supplemented diet for weaned piglets on up-regulating mRNA expression of *OCLN* (*P* = 0.08) and down-regulating (*P* < 0.05) mRNA expressions of *TLR-9* and *NF-κB* in the ileum of weaned piglets (Fig. 5A and C). Weaned piglets from the CA-CON or CA-CA group had lower mRNA expressions of *TLR-9* and *NF-κB* in the ileum compared with weaned piglets from the CON-CON or CON-CA group (*P* < 0.05, Fig. 5C).

## Discussion

In this study, the litter size at farrowing was not markedly affected by maternal CA supplementation, which is reasonable given the short period of CA supplementation, and the number of piglets per litter is generally determined by early ovulation rate and conception [24, 25]. However, maternal CA supplementation tended to increase the growth rate of suckling piglets, which may be related to improved milk composition, as the concentrations of fat in colostrum, IgG, and IgM in milk were increased by CA supplementation. Similarly, maternal supplementation with plant-derived bioactive components like *Lonicera flos* and *Sucutellaria baicalensis* has been found to improve milk composition [26]. As the sole nutrient source, colostrum and milk intake are vital for piglet growth and development [27, 28]. Milk fat provides energy for thermoregulation and body growth in newborn piglets [29], while milk immunoglobulins help develop passive immunity [30]. In this study, the optimized milk composition may reflect the beneficial effects of CA on sow health status and mammary gland development, as sow health is closely linked to milk composition [31]. Studies have reported that CA could reduce cytokine production (e.g., IL-6 and TNF-α) by regulating the NF-κB pathway [32]. Thus, the levels of IgG and IgM may be associated with CA-induced changes in the inflammatory environment of the mammary gland. Additionally, systemic oxidative stress in sows may damage mammary glands and reduce both milk quality and yield [33], while antioxidant supplementation has been shown to improve the redox status and milk composition of sows [34].

In this study, supportively, sows fed the CA diet had higher plasma activities of GSH-Px and T-SOD, indicating that the improved health status of sows fed with CA may partially account for the enhancement of milk composition. Acting as an agonist for nuclear factor erythroid 2-related factor 2 (Nrf2), CA facilitates the entry of Nrf2 into the cell nucleus, where it binds to antioxidant response elements, resulting in increased expression of antioxidant genes and enzymes [35, 36]. Additionally, oxidative stress may negatively impact the growth performance of piglets [37]. The excessive accumulation of ROS disrupts systemic oxidative homeostasis, inducing peroxidative damage to subcellular organelles and extracellular matrix components. The piglet intestine is primarily affected by ROS, leading to the destruction of intestinal structure, microbial imbalance, and nutrient absorption disorders, and ultimately results in lower feed intake and body weight gain, as well as systemic metabolic imbalances [38]. Antioxidant enzymes, such as GSH-Px, T-SOD, and CAT, could help reduce the levels of toxic ROS [39]. Our previous study also found a positive correlation between plasma and ileal antioxidant enzyme levels and the growth performance of piglets [40]. In this study, the higher activities of antioxidant enzymes and growth rates were observed in piglets at the end of suckling from CA-supplemented sows.

In mammals, the small intestine is crucial for nutrient digestion and absorption [41], and it also defends against pathogen invasion [42]. Claudin-1, ZO-1, and OCLN are key to the structure and function of epithelial tight junctions [43]. TLR recognizes endotoxins from Gram-negative bacteria, activating the MyD88/NF-кβ pathways and promoting cytokine expression, such as TNF-α, IL-1β, and IL-6 [44]. In this study, piglets from CA-supplemented sows had lower expressions of *TLR-4* and *TNF-α* and higher expressions of *OCLN* and *ZO-1* in the ileal mucosa at the end of suckling, indicating maternal CA supplementation benefits offspring’s intestinal function, though the underlying mechanism requires further investigation.

Diet significantly influences gut microbiota composition, function, and diversity [45]. In our study, maternal CA supplementation showed limited effects on sow gut microbiota at the end of lactation. However, LEfSe analysis revealed *Escherichia-Shigella* as the dominant bacterial species in CON sows. Notably, *Escherichia-Shigella* can be transmitted from the sows and colonized in the intestines of newborn piglets [46]. As a member of the Gram-negative Enterobacteriaceae family, *Shigella* causes severe watery diarrhea in hosts [47] by crossing the intestinal epithelium, triggering macrophage death, and disrupting the large intestinal epithelium [48]. The gut microbiota is crucial for maintaining intestinal homeostasis in newborn piglets [49]. Maternal sources like the vagina, milk, skin, and feces provide initial bacterial communities for piglet gut colonization [46]. In our study, maternal CA supplementation significantly increased Firmicutes abundance and the Firmicutes/Bacteroides ratio in piglet colonic digesta at the end of suckling. Firmicutes are more efficient than Bacteroidetes at energy extraction from food, potentially enhancing calorie absorption and weight gain [50]. Additionally, maternal CA supplementation significantly reduced *Fusobacterium* and *Sutterella* levels in piglet colonic digesta at PND 24. These pathogens are associated with severe intestinal damage [51, 52]. The reduction in pathogenic bacteria through maternal CA supplementation may be programmed to positively affect post-weaning growth and intestinal health. Consistently, maternal CA supplementation improved the post-weaning F:G, diarrhea scores, nutrient digestibility, sucrase activity, intestinal morphology, and mRNA expressions of anti-inflammatory-related and intestinal barrier genes in weaned piglets. Similar beneficial effects on intestinal health and development have been observed with maternal live yeast supplementation [53].

Post-weaning CA supplementation also tended to reduce diarrhea scores from d 14 to 21, which was consistent with previous results [54, 55]. Weaning stress can cause systemic oxidative stress and inflammation [56], leading to intestinal dysfunction and growth reduction in weaned piglets [57, 58]. However, CA enhances antioxidant defenses against ROS via Nrf2 activation [59] and reduces inflammation by inhibiting the NF-кB pathway and cytokine expression in rodent animals [60]. In this study, likewise, post-weaning CA supplementation improved plasma antioxidant enzyme activities and mRNA expressions of *SOD* and *IL-10* in the ileal mucosa, while decreasing inflammation-related markers such as plasma IL-1β concentration and mRNA expressions of *TLR-9* and *NF-кB* in the ileal mucosa, which are key factors in alleviating diarrhea and intestinal injury.

## Conclusions

Maternal CA supplementation during d 107 of gestation to d 24 of lactation tended to increase the growth rate and weaning weight of suckling piglets, which may be related to the improvement of CA on antioxidant capacity and milk composition. Moreover, maternal CA supplementation or post-weaning CA supplementation tended to improve diarrhea scores, along with the improved nutrient digestibility, redox status and intestinal function-related parameters of weaned piglets.

## Data Availability

The data used during the current study are available from the corresponding author upon reasonable request.

## Abbreviations

ADFI: Average daily feed intake
ADG: Average daily gain
ALT: Alanine aminotransferase
APA: Aminopeptidases A
APN: Aminopeptidases N
AST: Aspartate amino transferase
ATTD: Apparent total tract digestibility
C3: Complement C3
CA: Cinnamaldehyde
CAT: Catalase
CLDN1: Claudin 1
CP: Crude protein
CRP: C-reactive protein
DM: Dry matter
EE: Ether extract
FG: The ratio of average daily feed intake to average daily gain
GE: Gross energy
GGT: Gamma-glutamyl transpeptidase
GPX1: Glutathione peroxidase 1
GSH-Px: Glutathione peroxidase
IgG: Immunoglobulin G
IgM: Immunoglobulin M
IL-1β: Interleukin-1β
IL-6: Interleukin-6
IL-10: Interleukin-10
MDA: Malondialdehyde
MyD88: Myeloid differentiation factor 88
NEFA: Non-esterified fatty acid
NF-κB: Nuclear factor kappa B
OCLN: Occludin
PCoA: Principal coordinate analysis
PND: Post-natal day
PWD: Post-weaned day
SCFA: Short-chain fatty acid
SOD1: Superoxide dismutase 1
SOD2: Superoxide dismutase 2
TBA: Total bile acid
TG: Total cholesterol
TLR-4: Toll-like receptor 4
TLR-9: Toll-like receptor 9
TNF-α: Tumor necrosis factor-α
T-SOD: Total superoxide dismutase
VCR: The ratio of villus height to crypt depth
ZO-1: Zonula occludens-1
